# Molecular mechanisms and therapeutic progress in atherosclerosis: bridging immune inflammation and precision medicine

**DOI:** 10.3389/fimmu.2025.1737662

**Published:** 2026-01-05

**Authors:** Yimin Han, Hongyi Xu, Xinlei Yao, Zhanzhan Li, Chuli Zhu, Xia Li, Bingqian Chen, Hualin Sun

**Affiliations:** 1 Jiangsu Key Laboratory of Tissue Engineering and Neuroregeneration, Key Laboratory of Neuroregeneration of Ministry of Education, Co-innovation Center of Neuroregeneration, Medical School of Nantong University, Nantong University, Nantong, Jiangsu, China; 2 Department of Orthopedics, Changshu Hospital Affiliated to Soochow University, First People’s Hospital of Changshu City, Changshu, Jiangsu, China

**Keywords:** atherosclerosis, immune inflammation, immunomodulatory vaccines, NLRP3 inflammasome, precision medicine

## Abstract

Atherosclerosis, a chronic vascular disease characterized by lipid-driven inflammation and arterial wall remodeling, remains the leading cause of cardiovascular morbidity and mortality. This review aims to systematically summarize recent advances in our understanding of its multidimensional pathogenesis and the corresponding evolution of therapeutic strategies. We focus on the intricate crosstalk between endothelial dysfunction, dyslipidemia, and immune-inflammatory activation, which collectively drive disease progression. Key mechanisms discussed include metabolic reprogramming of immune cells, phenotypic switching of vascular smooth muscle cells, and novel modes of programmed cell death. Beyond conventional lipid-lowering approaches, we highlight emerging therapeutic avenues such as immunomodulatory vaccines, RNA-based therapeutics, and biodegradable stents with inherent anti-ferroptotic properties. Furthermore, we explore the potential of modernized traditional Chinese medicine formulations that target multiple pathways. Looking forward, we conclude that integrating multi-omics data, single-cell technologies, and artificial intelligence is pivotal for advancing precision medicine. This integration will enable the development of individualized risk prediction models and targeted interventions, ultimately bridging the gap between molecular mechanisms and effective clinical management to overcome current bottlenecks in atherosclerosis prevention and treatment.

## 1 Introduction

Arteriosclerosis is a progressive pathological process and serves as the common pathological basis for various cardiovascular and cerebrovascular diseases. The development of arteriosclerosis is a prolonged and insidious process. Key risk factors include advanced age, hypertension, diabetes, dyslipidemia, smoking, and chronic kidney disease (1, 2). Globally, it represents the leading cause of death and disability, imposing a substantial socioeconomic burden (3). Notably, based on the size of affected vessels and pathological features, it is primarily classified into atherosclerosis, arteriolosclerosis, medial calcification (Monckeberg’s medial calcific sclerosis), and fibromuscular intimal hyperplasia (4, 5). Among these, atherosclerosis is the most extensively studied and clinically significant. Its typical pathological features involve the accumulation of lipids, inflammatory cells, and fibrous tissue in the intima of large and medium-sized arteries, forming atheromatous plaques. This ultimately leads to luminal stenosis, plaque rupture, and acute thrombotic events. Elucidating its pathogenesis and developing effective prevention and treatment strategies are crucial for reducing the global burden of cardiovascular diseases.

Current research increasingly adopts a systemic and multi-factorial perspective to elucidate its pathological processes. The traditional “lipid infiltration” and “endothelial injury response” hypotheses have expanded into a complex regulatory network involving chronic inflammation, immune responses, cellular senescence, metabolic disturbances, and vascular calcification. Regarding immune-inflammatory mechanisms, both innate and adaptive immune systems are deeply involved throughout arteriosclerosis progression. At the level of metabolism and cellular dysfunction, multiple mechanisms drive disease progression. On one hand, intracellular labile iron overload caused by iron homeostasis imbalance exacerbates oxidative stress (6). On the other hand, dysregulated phosphate metabolism leading to hyperphosphatemia, promotes vascular calcification and a senescence-like phenotype. Furthermore, phenotypic switching of vascular smooth muscle cells is a key step in neointima formation (7). Meanwhile, histone deacetylases, as crucial epigenetic regulators, participate in disease progression by influencing inflammation, proliferation, and migration of vascular endothelial and smooth muscle cells (2).

Significant progress has been made in prevention and treatment strategies in recent years, particularly through lifestyle interventions such as regular physical activity (1). Pharmacologically, although intensive statin-based lipid-lowering remains foundational, significant residual cardiovascular risk persists even after achieving LDL cholesterol targets (8, 9). Beyond conventional drugs, immune-based strategies have shown promise in preclinical models (10). Interventional therapy also demonstrate considerable application potential (11). In summary, arteriosclerosis management is increasingly moving toward multi-target, individualized, and comprehensive interventions. Despite notable advances, several challenges remain. First, as a highly heterogeneous disease, deeper molecular and cellular-level analyses of heterogeneity sources are urgently needed. Second, balancing anti-inflammatory effects with infection risks from immunosuppression presents a practical translational challenge. Third, there are a lack of effective early identification and intervention methods, limiting timely disease progression blocking. Finally, many treatments effective in animal models fail to show comparable efficacy in human trials (12). Developing more predictive experimental models is essential for clinical translation.

This review aims to: First, integrate multidimensional pathogenesis—going beyond traditional frameworks to include lipid metabolism disorders, chronic inflammation, immune responses, cellular senescence, epigenetic regulation, vascular calcification, and metabolic abnormalities (e.g., iron, phosphate)—and construct a more holistic molecular network. Second, it will distinguish common and specific features of different pathological types, clearly differentiating atherosclerosis, arteriolosclerosis, and medial calcification (4), and explore their unique molecular drivers and converging pathways to advance precision medicine. Third, it will assess the potential and limitations of existing and emerging strategies—from lifestyle interventions and conventional drugs to novel therapies (9–11)—and analyze challenges and failures in clinical trials to inform future studies. Finally, it will outline future research directions and translational pathways, emphasizing the use of single-cell sequencing, spatial transcriptomics, and multi-omics integration to dissect cellular heterogeneity and intercellular communication in lesions. It also calls for increased focus on early biomarker discovery and interventions targeting initial disease events, ultimately contributing to precise prevention, early diagnosis, and effective treatment of arteriosclerosis.

## 2 Molecular mechanisms of atherosclerosis

**Figure 1:**
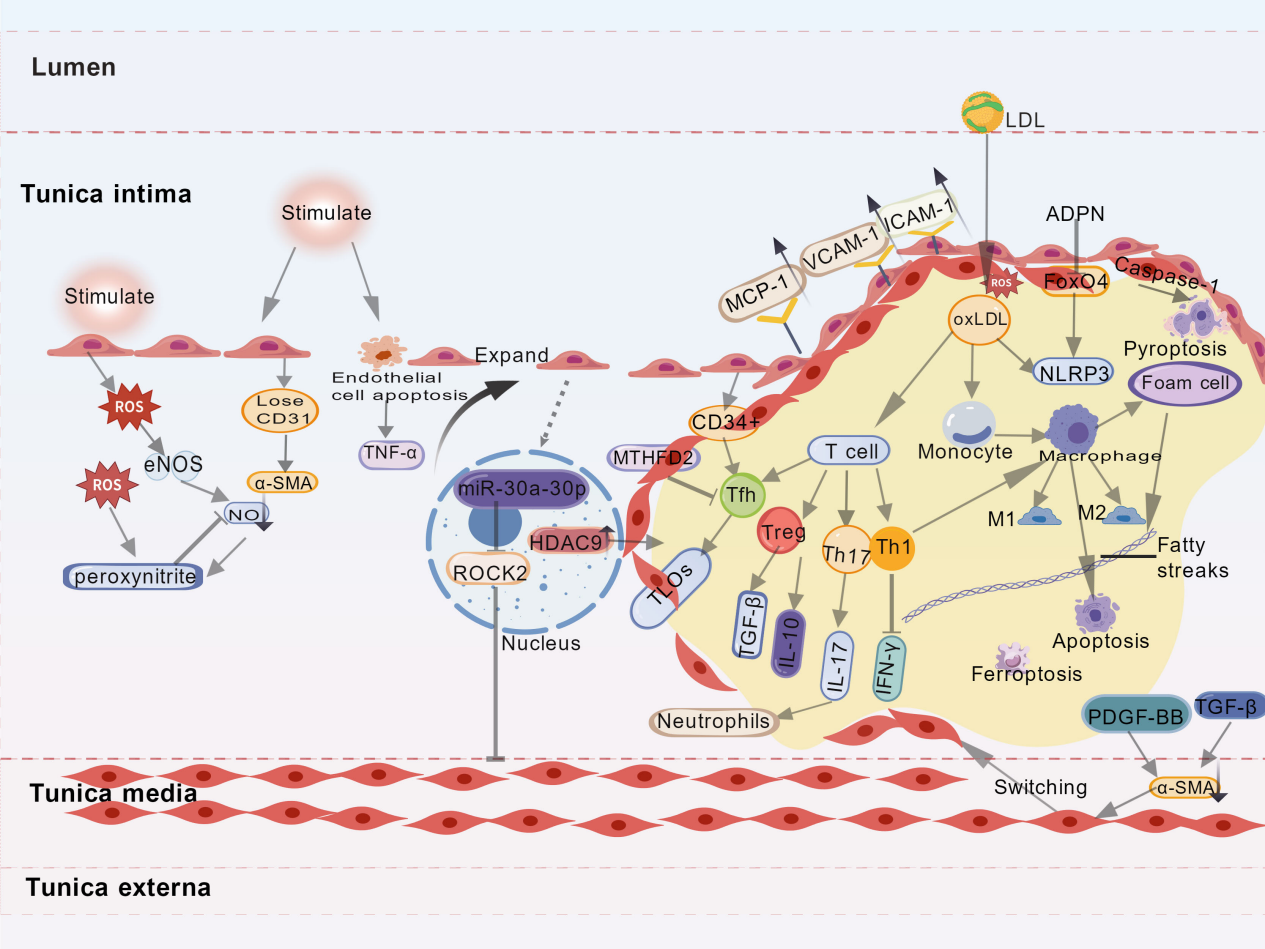
Molecular mechanisms of atherosclerosis. This figure illustrates key molecular mechanisms in arteriosclerosis, focusing on atherosclerosis. It involves endothelial dysfunction, lipid dysregulation, immune-inflammatory activation, cell death, and vascular smooth muscle cell (VSMC) phenotypic switching. Endothelial injury leads to increased reactive oxygen species (ROS), which reduce nitric oxide (NO) availability and promote oxidative damage. Endothelial cells lose CD31 and express α-smooth muscle actin (α-SMA), facilitating monocyte adhesion and inflammation. MicroRNAs such as miR-30a-30p modulate vascular remodeling via epigenetic regulators including HDAC9 and ROCK2. Lipid deposition occurs as LDL enters the intima and oxidizes into oxLDL, triggering expression of adhesion molecules and monocyte recruitment. Macrophages ingest oxLDL, forming foam cells, which undergo pyroptosis, apoptosis, or ferroptosis. Macrophage polarization (M1/M2) further shapes inflammation. Immune cells—such as Treg, Th17, and Th1—secrete cytokines (e.g., TGF-β, IL-17, IFN-γ) that regulate plaque progression. Factors like PDGF-BB and TGF-β drive VSMC phenotypic switching, enhancing α-SMA expression and vascular remodeling, thereby advancing atherosclerotic lesions.

Atherosclerosis serves as the primary pathological basis of cardiovascular diseases. Its development involves complex interactions among various cells and molecular mechanisms. Recent studies indicate that endothelial dysfunction, dyslipidemia, immune-inflammatory responses, phenotypic switching of vascular smooth muscle cells, programmed cell death, and epigenetic regulation collectively form the core pathophysiological network of atherosclerosis (**Figure 1**). Notably, emerging research directions such as metabolic reprogramming and immunometabolic regulation have further expanded our understanding of the disease mechanisms.

### 2.1 Endothelial cell dysfunction and activation

Atherosclerosis is a chronic inflammatory vascular disease that begins with endothelial cell dysfunction. Endothelial cells act as the first line of defense in the vascular wall, playing a key role in maintaining vascular homeostasis. They regulate vascular tone, suppress inflammation, and prevent thrombosis. However, under risk factors such as hemodynamic changes, hypercholesterolemia, hypertension, diabetes, and smoking, endothelial cells transition from an anti-inflammatory and anticoagulant state to a pro-inflammatory and pro-thrombotic state. This process, termed endothelial dysfunction and activation, represents the initial step in atherosclerosis (13). Here, we explore the molecular mechanisms of endothelial dysfunction in atherosclerosis, focusing on reduced nitric oxide bioavailability, upregulation of adhesion molecules and chemokines, as well as endothelial cell apoptosis and endothelial-mesenchymal transition.

#### 2.1.1 Endothelial nitric oxide synthase uncoupling and nitric oxide deficiency

Nitric oxide (NO) is a critical signaling molecule produced by endothelial cells. It mediates vasodilation, anti-inflammatory effects, and anti-proliferative functions. Under physiological conditions, endothelial nitric oxide synthase (eNOS) catalyzes the production of NO from L-arginine to maintain vascular homeostasis. However, under atherosclerotic risk factors, elevated oxidative stress induces eNOS uncoupling. Uncoupled eNOS not only reduces NO synthesis but also generates ROS such as superoxide anions, further exacerbating oxidative stress (13). Additionally, ROS react with NO to form peroxynitrite, significantly diminishing NO bioavailability. This promotes vasoconstriction, inflammatory cell infiltration, and smooth muscle cell proliferation. Studies show that in diabetes-associated atherosclerosis, hyperglycemia exacerbates eNOS dysfunction through enhanced oxidative stress, accelerating disease progression (14). Recent research also reveals that RIPK1 in endothelial cells modulates protein synthesis pathways affecting NO-related signaling, and RIPK1 deficiency exacerbates vascular stenosis in transplant-associated atherosclerosis (13). Therefore, restoring NO bioavailability has become a key strategy for atherosclerosis prevention and treatment. Targeting eNOS function and oxidative stress may offer novel therapeutic approaches.

#### 2.1.2 Upregulation of adhesion molecules and chemokines

Activated endothelial cells highly express adhesion molecules and chemokines, promoting the adhesion and migration of immune cells such as monocytes and lymphocytes into the subendothelial space. This process is central to atherosclerotic plaque formation. Vascular cell adhesion molecule-1 (VCAM-1) and intercellular adhesion molecule-1 (ICAM-1) are key proteins mediating initial immune cell adhesion to endothelial cells. Their expression is significantly upregulated by inflammatory cytokines like tumor necrosis factor-α (TNF-α) (15). Meanwhile, chemokines such as monocyte chemoattractant protein-1 (MCP-1) guide immune cell migration to lesion sites. Recent studies highlight the importance of the CCL21/CXCR3 axis in immune cell chemotaxis. In transplant atherosclerosis, CCL21 expressed by lymphatic endothelial cells binds to the CXCR3 receptor, recruiting T cells and dendritic cells to form tertiary lymphoid organ-like structures, thereby amplifying local immune responses (16). Single-cell RNA sequencing further confirms that the CCL21/CXCR3 axis is a key pathway regulating early immune cell infiltration. Neutralizing CCL21 or CXCR3 significantly attenuates atherosclerotic lesions (16). Additionally, long non-coding RNAs such as HOXA11-AS regulate inflammatory cytokine expression via the PI3K/AKT pathway, promoting diabetes-associated atherosclerosis (17). Thus, targeting adhesion molecules and chemokine signaling may provide effective strategies to inhibit immune cell infiltration and delay atherosclerosis progression.

#### 2.1.3 Endothelial cell apoptosis and endothelial-mesenchymal transition

Persistent pathological stimuli induce endothelial cell apoptosis, disrupting endothelial integrity, increasing vascular permeability, and exposing the subendothelial matrix. This accelerates lipid deposition and inflammatory responses. Apoptotic endothelial cells also release factors such as transforming growth factor-β (TGF-β), further promoting vascular remodeling (18). More importantly, endothelial cells can undergo endothelial-mesenchymal transition (EMT), transforming into migratory and proliferative mesenchymal cells that contribute to neointima formation. During EMT, endothelial cells lose specific markers like CD31 and acquire mesenchymal markers such as α-smooth muscle actin. In transplant atherosclerosis models, endothelial apoptosis induces TGF-β signaling-dependent EMT, leading to the accumulation of recipient-derived smooth muscle-like cells in the neointima (18). Furthermore, the long non-coding RNA MAGOH-DT is upregulated in TNF-α and high glucose-induced EMT and promotes the development of atherosclerotic occlusion by regulating TGF-β2 expression (19). Another mechanism involves microbial infections; for example, Helicobacter pylori’s CagA protein can enter endothelial cells via exosomes, activate STAT3 signaling, and induce inflammation and EMT, thereby accelerating atherosclerosis (20). Therefore, inhibiting endothelial apoptosis and EMT may help maintain endothelial integrity and slow atherosclerosis progression.

In summary, endothelial dysfunction and activation are central to atherosclerosis development. They involve interconnected molecular mechanisms such as reduced NO bioavailability, upregulation of adhesion molecules and chemokines, and EMT. These processes form a complex regulatory network that collectively drives disease progression. Future research should focus on specifically targeting these pathways, for example, by modulating RIPK1 signaling (13) or inhibiting the CCL21/CXCR3 axis (16), to develop novel prevention and treatment strategies. Additionally, interventions targeting EMT and oxidative stress hold promise for opening new avenues in atherosclerosis therapy.

### 2.2 Lipid metabolism disorders and modifications

Atherosclerosis is a chronic inflammatory vascular disease, and its progression is closely linked to lipid metabolism disorders. The retention and modification of lipids, particularly low-density lipoprotein (LDL), within the arterial wall represent a central event in atherogenesis. This process involves not only classical mechanisms such as lipoprotein oxidation and foam cell formation but also emerging perspectives like metabolomics and organelle function. Therefore, elucidating these mechanisms is essential for a comprehensive understanding of atherosclerosis.

#### 2.2.1 Lipoprotein retention and oxidative modification

After low-density lipoprotein (LDL) crosses the damaged endothelium into the arterial intima, it is retained by the extracellular matrix and undergoes oxidative modification to form oxidized LDL (OxLDL). This process is mainly mediated by ROS, including superoxide anion and hydrogen peroxide (21). OxLDL is not only a key component of atherosclerotic plaques but also exerts multiple pathological effects that drive disease progression. First, OxLDL is recognized by scavenger receptors, such as SR-A1 and CD36, on macrophages. This leads to unregulated intracellular lipid uptake and foam cell formation (22, 23). Unlike the LDL receptor, which is regulated by negative feedback, scavenger receptors are not controlled by intracellular cholesterol levels, resulting in excessive lipid accumulation. Foam cells are markers of early atherosclerotic lesions. Their accumulation forms fatty streaks, and they release various inflammatory factors and chemokines, such as MCP-1 and TNF-α, thereby amplifying local inflammation (24). Thus, foam cell accumulation is a critical link between lipid metabolism disorders and inflammatory responses. Second, OxLDL has notable pro-inflammatory and cytotoxic effects. It activates endothelial cells and promotes the expression of adhesion molecules, including VCAM-1 and ICAM-1. This enhances the adhesion and migration of monocytes and T lymphocytes (25). Additionally, OxLDL induces vascular smooth muscle cells (SMCs) to migrate from the media to the intima and proliferate, contributing to fibrous cap formation. Moreover, OxLDL cytotoxicity can cause endothelial cell apoptosis and necrosis, worsening intimal injury and plaque instability (26). Therefore, OxLDL promotes atherosclerosis through multiple synergistic mechanisms.

#### 2.2.2 Lipid metabolism disorders

In recent years, new technologies have deepened our understanding of lipid metabolism disorders in atherosclerosis. Significant advances have been made in metabolomics and organelle interactions. Mendelian randomization studies provide evidence for causal relationships between specific blood metabolites and atherosclerosis. For example, certain glycerophospholipids and bile acid metabolites are significantly associated with peripheral atherosclerosis (27). Specifically, elevated lysophosphatidylcholine levels may contribute to atherosclerosis by promoting endothelial inflammation and monocyte activation. These findings reveal potential biomarkers and offer new insights into disease mechanisms through metabolic pathways. They also suggest that modulating specific metabolic pathways could be an effective intervention strategy, highlighting the central role of metabolomics in elucidating disease mechanisms. Concurrently, mitochondria-associated endoplasmic reticulum membrane (MAM) dysfunction is gaining attention. MAM is a dynamic contact zone between mitochondria and the endoplasmic reticulum, and it plays a key role in regulating intracellular calcium homeostasis and lipid metabolism. Recent studies indicate that MAM dysfunction impairs vascular cell function by disrupting calcium homeostasis and lipid metabolism, and it is linked to the progression of transplant atherosclerosis (28). In atherosclerosis, aberrant MAM stability can trigger endoplasmic reticulum stress and mitochondrial calcium overload. This leads to excessive mitochondrial ROS production and cell apoptosis. Furthermore, MAM dysregulation affects cholesterol synthesis and transport, exacerbating intracellular lipid accumulation (29). For instance, altered expression of MAM-related proteins like FUNDC1 and IP3R is associated with lipid metabolism disorders and phenotypic switching in vascular smooth muscle cells. Thus, maintaining MAM functional homeostasis is vital for vascular health. These advances collectively drive the in-depth study of atherosclerosis mechanisms.

### 2.3 Activation of innate and adaptive immune responses

**Figure 2:**
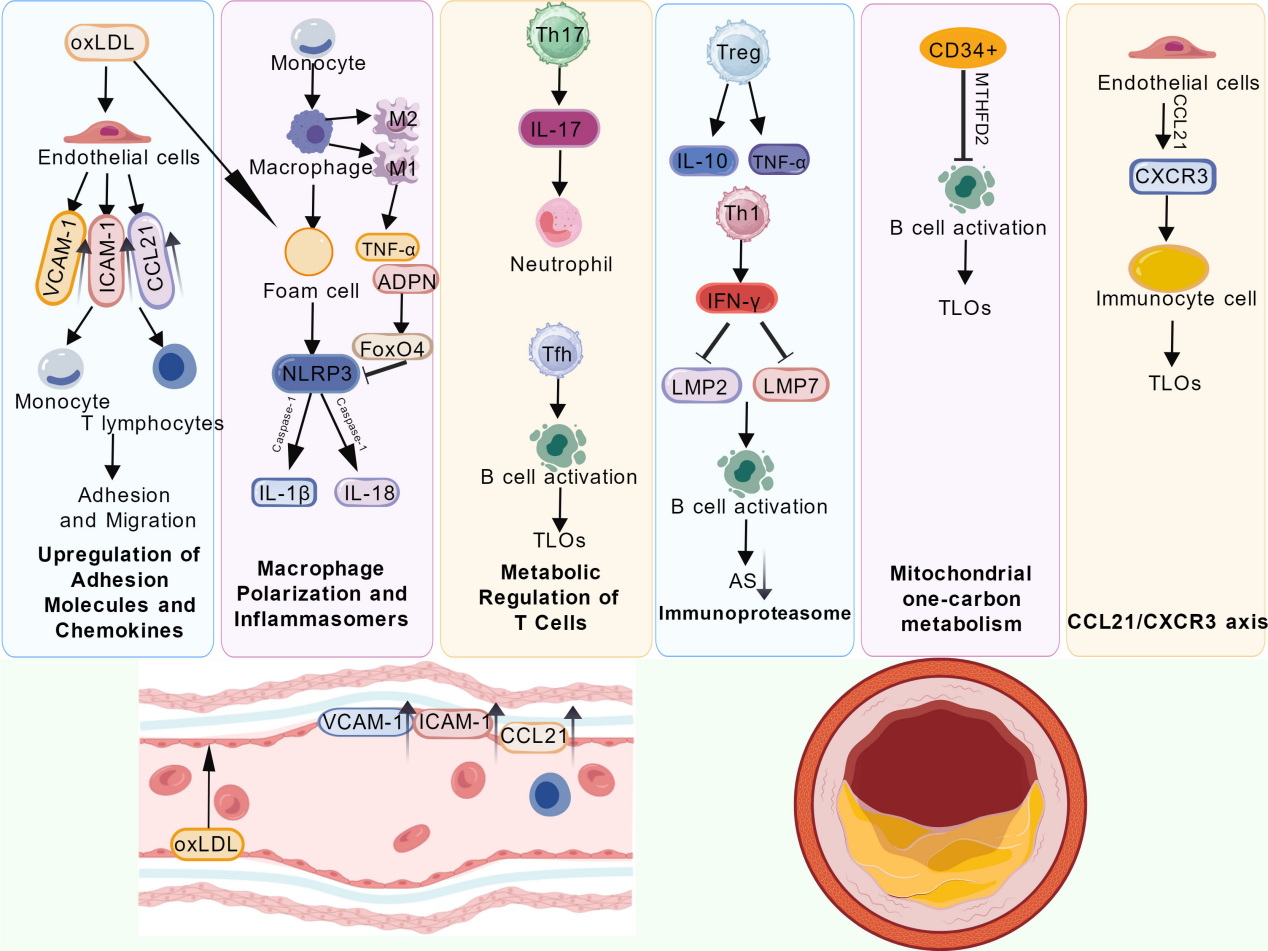
Immune-inflammatory network in atherosclerosis. This schematic depicts the regulatory network of immune inflammation and metabolic reprogramming in atherosclerosis, tracing the progression from vascular injury to plaque formation. Stimulated by oxLDL, endothelial cells upregulate adhesion molecules (VCAM-1, ICAM-1) and chemokines (CCL21), promoting monocyte and T-cell recruitment into the vessel wall. Monocytes differentiate into macrophages, which polarize into M1 (pro-inflammatory, releasing TNF-α) or M2 (anti-inflammatory) subtypes. Upon oxLDL uptake, they form foam cells. The FoxO4-regulated NLRP3 inflammasome activates Caspase-1, triggering IL-1β and IL-18 release, with adiponectin (ADPN) involved in modulation. In T-cell metabolic regulation, Th17 cells secrete IL-17 to activate neutrophils, while Tfh cells assist B-cell activation, fostering tertiary lymphoid organ (TLO) formation. Tregs modulate Th1-derived IFN-γ via IL-10 and TNF-α. IFN-γ induces immunoproteasome subunits LMP2/LMP7, further supporting B-cell activation and TLO development. CD34+ cells influence mitochondrial one-carbon metabolism via MTHFD2, aiding B-cell activation and TLO formation. Finally, the CCL21/CXCR3 axis mediates immune cell recruitment and TLO development, accelerating disease progression.

Atherosclerotic plaques are not merely lipid accumulations but highly complex immune microenvironments. Innate and adaptive immune responses interact via intricate cellular and molecular networks, collectively driving disease onset and progression (**Figure 2**). Deeply elucidating these immune mechanisms is essential for developing novel prevention and treatment strategies.

#### 2.3.1 Macrophage polarization and inflammasomes

In early atherosclerosis, monocyte infiltration into the vascular intima and their differentiation into macrophages are central events. Macrophages are heterogeneous and highly plastic, primarily categorized into pro-inflammatory M1 and anti-inflammatory M2 types. M1 macrophages are characterized by high expression of markers such as inducible nitric oxide synthase (iNOS) and the production of pro-inflammatory cytokines like IL-1β, IL-6, and TNF-α, which exacerbate plaque inflammation and instability. In contrast, M2 macrophages, identified by markers like the mannose receptor (CD206) and the production of anti-inflammatory cytokines such as IL-10 and TGF-β, promote tissue repair, enhance efferocytosis, and contribute to plaque stability (30). The M2 phenotype is further divided into subtypes (M2a, M2b, M2c, M2d) with nuanced functions. Furthermore, other distinct macrophage phenotypes are identified in atherosclerotic plaques. Intraplaque hemorrhage induces the Mhem phenotype, characterized by high expression of heme oxygenase-1 (HO-1) and efficient iron handling, which confers protection against oxidative injury and exhibits anti-atherogenic properties (31, 32). The CXCL4-induced M4 macrophage expresses a unique transcriptome with lower scavenger receptor activity but higher expression of pro-inflammatory mediators like IL-6, TNF-α, matrix metalloproteinase-7 (MMP7), and S100A8, contributing to plaque vulnerability (33, 34). Exposure to oxidized phospholipids drives the formation of Mox macrophages via the Nrf2 pathway, defined by the upregulation of antioxidant enzymes like heme oxygenase-1 (HMOX1), thioredoxin reductase 1 (Txnrd1), and sulfiredoxin-1 (Srxn-1), yet their net role in atherosclerosis progression remains complex (35). Therefore, the balance among these diverse macrophage subtypes is critical for disease progression and regression.

Moreover, NLRP3 inflammasome activation serves as a key amplifier connecting metabolic disorders to atherosclerosis inflammation. When macrophages ingest oxidized LDL to become foam cells or detect danger signals like cholesterol crystals, they assemble the inflammasome. This activates caspase-1, which cleaves pro-IL-1β and pro-IL-18 into mature cytokines. These cytokines directly injure the vascular endothelium and recruit neutrophils, creating a positive feedback loop for inflammation. Adiponectin, an important adipokine, counteracts this by inhibiting transcription factor FoxO4, thus reducing NLRP3-mediated endothelial pyroptosis and exerting anti-atherosclerotic effects (36). Therefore, targeting the inflammasome pathway could be an effective intervention. Additionally, epigenetic regulation, especially m6A RNA methylation, is crucial in macrophage polarization. Research shows that the m6A demethylase ALKBH5 reduces m6A modification on integrin β1 mRNA, enhancing its stability. This promotes M2 polarization and mitigates lower extremity atherosclerosis (37). This implies that targeting RNA epigenetics to modulate immune cell function may offer novel therapeutic avenues.

#### 2.3.2 Metabolic regulation of T cells

Adaptive immune responses, especially those mediated by T cells, are central to shaping the plaque immune microenvironment. CD4^+^ T helper (Th) cell subsets play dual roles in atherosclerosis, defined by their distinct cytokine profiles, transcription factors, and functional impacts.

Pro-atherogenic Th1 cells are characterized by the master transcription factor T-bet and secrete high levels of interferon-γ (IFN-γ) (38). IFN-γ activates pro-inflammatory macrophages (M1), inhibits collagen production by vascular smooth muscle cells (VSMCs), and upregulates endothelial adhesion molecules, collectively promoting plaque inflammation and instability (38, 39). Conversely, Th2 cells, defined by GATA3 expression, produce cytokines such as IL-4, IL-5, and IL-13. Their role in atherosclerosis is context-dependent but is often associated with IgG1 and IgE class-switching in B cells, which can be pro-atherogenic (38, 39). Th17 cells express the transcription factor RORγt and secrete IL-17A, IL-17F, and IL-22. They recruit neutrophils, promote vascular inflammation, and exacerbate plaque progression (38, 40). In contrast, regulatory T cells (Tregs) are protective, defined by the expression of the transcription factor Foxp3. They secrete anti-inflammatory cytokines like IL-10 and TGF-β, which suppress effector T cell activation, modulate macrophage polarization, and help maintain immune tolerance and plaque stability (41, 42).

Recent years have seen expanded knowledge of T cell subsets and their specialized niches. A key population is the T follicular helper (Tfh) cell, which is essential for driving B cell responses in germinal centers (GCs) and extrafollicular areas. Tfh cells are defined by the expression of the transcription factor BCL6, the chemokine receptor CXCR5, surface markers such as inducible costimulator (ICOS) and programmed cell death-1 (PD-1), and the secretion of signature cytokines like IL-21 (43). Tfh cell polarization is driven by specific cytokines: IL-6 in mice, and IL-12, TGF-β, and activin A in humans (43). Furthermore, Tfh cells can adopt specialized subsets mirroring other Th lineages: Tfh1 (producing IFN-γ), Tfh2 (producing IL-4), Tfh13 (producing IL-13), and Tfh17 (producing IL-17), which skew B cell antibody class-switching toward specific isotypes (e.g., IgG2a/c, IgG1, IgE) (43, 44). For instance, in transplant atherosclerosis models, host-derived CD34^+^ lineage cells differentiate into Tfh cells through mitochondrial one-carbon metabolism. Research confirms that MTHFD2, a key enzyme in mitochondrial one-carbon metabolism, acts as a metabolic checkpoint in this differentiation. Inhibiting MTHFD2 reduces Tfh cell numbers and tertiary lymphoid organ formation, thereby inhibiting vascular remodeling (45). This links metabolic reprogramming, specific T cell differentiation, and vascular inflammation.

Beyond CD4^+^ T cells, cytotoxic CD8^+^ T cells also promote plaque instability. They recognize antigens presented on MHC class I and exert effector functions by releasing cytolytic molecules like perforin and granzymes, directly killing antigen-presenting cells or vascular wall cells, and by producing pro-inflammatory cytokines such as IFN-γ, thereby exacerbating local tissue damage and inflammation (44, 46). The balance between these pro-atherogenic effector T cells (Th1, Th17, CD8+) and atheroprotective Tregs is crucial in determining disease outcome.

In conclusion, elucidating the functions, specific biomarkers, and metabolic regulation of T cell subsets offers new theoretical foundations for immune-based interventions in atherosclerosis, highlighting targets such as specific cytokine pathways, surface receptors, transcription factors, and metabolic enzymes.

#### 2.3.3 Immunoproteasome

The proteasome is the primary system for intracellular protein degradation. Its specialized form, the immunoproteasome, is induced by cytokines such as interferon-γ and enriched in immune cells. It participates in antigen processing and immune regulation. The immunoproteasome is a specialized variant of the proteasome, predominantly expressed in immune cells such as dendritic cells, macrophages, and lymphocytes, and induced by inflammatory cytokines such as interferon−γ. It plays a crucial role in antigen processing by generating peptides that bind to MHC class I molecules, thereby activating CD8^+^ T cells. Beyond its role in adaptive immunity, the immunoproteasome is involved in regulating various inflammatory signaling pathways, including NF−κB activation, which influences cytokine production, immune cell survival, and function (47). In the context of atherosclerosis, the chronic inflammatory milieu is closely linked to persistent immune cell activation. Studies have shown that the immunoproteasome is up−regulated in immune cells within atherosclerotic plaques, particularly in activated T cells and macrophages. Its role in T cell activation and differentiation suggests that the immunoproteasome may influence atherosclerotic progression by modulating T cell responsiveness. For instance, in models of transplant−associated atherosclerosis, inhibition of the immunoproteasome subunits LMP2 and LMP7 significantly reduced T cell activation and alloantibody production by plasma cells, thereby attenuating atherosclerosis development (48). Compared with broad proteasome inhibition, selectively targeting the immunoproteasome may offer a more specific immunomodulatory strategy, potentially reducing the risks associated with systemic immunosuppression. This makes the immunoproteasome a promising therapeutic target for chronic inflammatory diseases such as atherosclerosis. Future studies could further explore the efficacy of immunoproteasome inhibitors in atherosclerosis models and evaluate their potential synergy with existing immune−checkpoint−modulating strategies.

In summary, Atherosclerosis is fundamentally a chronic inflammatory process driven by innate and adaptive immune responses. It involves macrophage polarization, inflammasome activation, T cell subset balance and function, and immunoproteasome-mediated fine-tuning of immunity. These elements form a complex immune network. Recent research not only enhances our knowledge of classic pathways like the adiponectin-FoxO4-NLRP3 and ALKBH5-m6A-ITGB1 axes in macrophage function but also uncovers new mechanisms. These include CD34^+^ cell differentiation into Tfh cells via mitochondrial one-carbon metabolism driving transplant atherosclerosis, and the synergistic role of immunoproteasome subunits in chronic rejection. Future studies should integrate multi-omics and spatial biology to better understand the heterogeneity and dynamics of the plaque immune microenvironment. Developing inhibitors or agonists for key molecules such as NLRP3 inflammasome, ALKBH5, MTHFD2, or specific immunoproteasome subunits could lead to novel immune-based strategies for atherosclerosis, offering more precise and effective clinical treatments.

### 2.4 Vascular smooth muscle cells: phenotypic switching, proliferation, and migration

Atherosclerosis is a chronic vascular condition characterized by arterial wall thickening, hardening, and loss of elasticity. Its pathogenesis involves complex interactions among multiple cellular and molecular events. Vascular smooth muscle cells (VSMCs), as the primary cellular component of the medial layer, play a central role in this pathological process. Under normal physiological conditions, VSMCs maintain a contractile phenotype to regulate vascular tone; however, under pathological stimuli, they can undergo phenotypic switching from contractile to synthetic. This transition enables VSMCs to proliferate, migrate, secrete extracellular matrix components and inflammatory factors, thereby driving neointima formation and vascular remodeling (49). Consequently, a deeper understanding of the molecular mechanisms underlying VSMC phenotypic switching is essential for elucidating the progression of atherosclerosis.

#### 2.4.1 Molecular regulatory mechanisms of phenotypic switching

The phenotypic switching of VSMCs is precisely regulated by various factors and signaling pathways. For instance, under stimulation by inflammatory factors such as platelet-derived growth factor-BB (PDGF-BB) and TGF-β, the expression of contractile markers like α-smooth muscle actin and calponin is downregulated, while synthetic phenotype-related genes are activated. Studies demonstrate that phosphatidylinositol 3-kinase γ promotes VSMC phenotypic modulation through a SOX9-dependent mechanism, and its knockout significantly suppresses neointima formation in mouse models (49). At the non-coding RNA level, microRNA-30a-3p inhibits proliferation and migration by targeting Rho-associated coiled-coil containing protein kinase 2 (50). In contrast, miR-21 promotes phenotypic switching and pathological progression by activating the AKT and extracellular signal-regulated kinase 1/2 pathways (51). Furthermore, circular RNA CircHAT1 disrupts the balance between cell proliferation and apoptosis by regulating serine/arginine-rich splicing factor 1, thereby exacerbating atherosclerotic lesions (52). These findings collectively highlight the central role of epigenetic regulation in VSMC behavior. Simultaneously, metabolic reprogramming serves as a key driver of phenotypic switching. Under hypoxic or inflammatory conditions, enhanced glycolysis and mitochondrial dysfunction further promote the acquisition of the synthetic phenotype via the mTOR and HIF-1α signaling pathways (53). Therefore, targeting these signaling and metabolic pathways may offer novel strategic avenues for preventing and treating atherosclerosis.

#### 2.4.2 Origins and heterogeneity of VSMCs

Traditional views posited that neointimal VSMCs originated solely from *in situ* proliferation within the medial layer; however, lineage tracing studies have revised this perspective. In transplant atherosclerosis models, recipient-derived c-Kit+ progenitor cells migrate to the graft and differentiate into neointimal VSMCs (53). This process is regulated by the stem cell factor/c-Kit axis and its downstream HK-1-dependent metabolic reprogramming, underscoring the significant contribution of circulating progenitor cells to vascular lesion formation. Single-cell RNA sequencing studies further reveal substantial heterogeneity among VSMCs in atherosclerosis, including subsets expressing stem cell markers that may possess enhanced proliferative and differentiation potential (13). Phenotypic switching, proliferation, and migration of VSMCs represent core events in the initiation and progression of atherosclerosis, involving multi-layered regulation by growth factors, signaling pathways, non-coding RNAs, and metabolic networks. A thorough understanding of these mechanisms not only elucidates the pathological basis of atherosclerosis but also opens new directions for targeted therapies. Future research should focus more on the functional implications of VSMC heterogeneity and explore how to achieve precise interventions by modulating key nodes in phenotypic switching, thereby providing theoretical foundations and practical approaches for improving clinical outcomes in atherosclerosis patients.

### 2.5 Apoptosis, pyroptosis and ferroptosis

**Figure 3:**
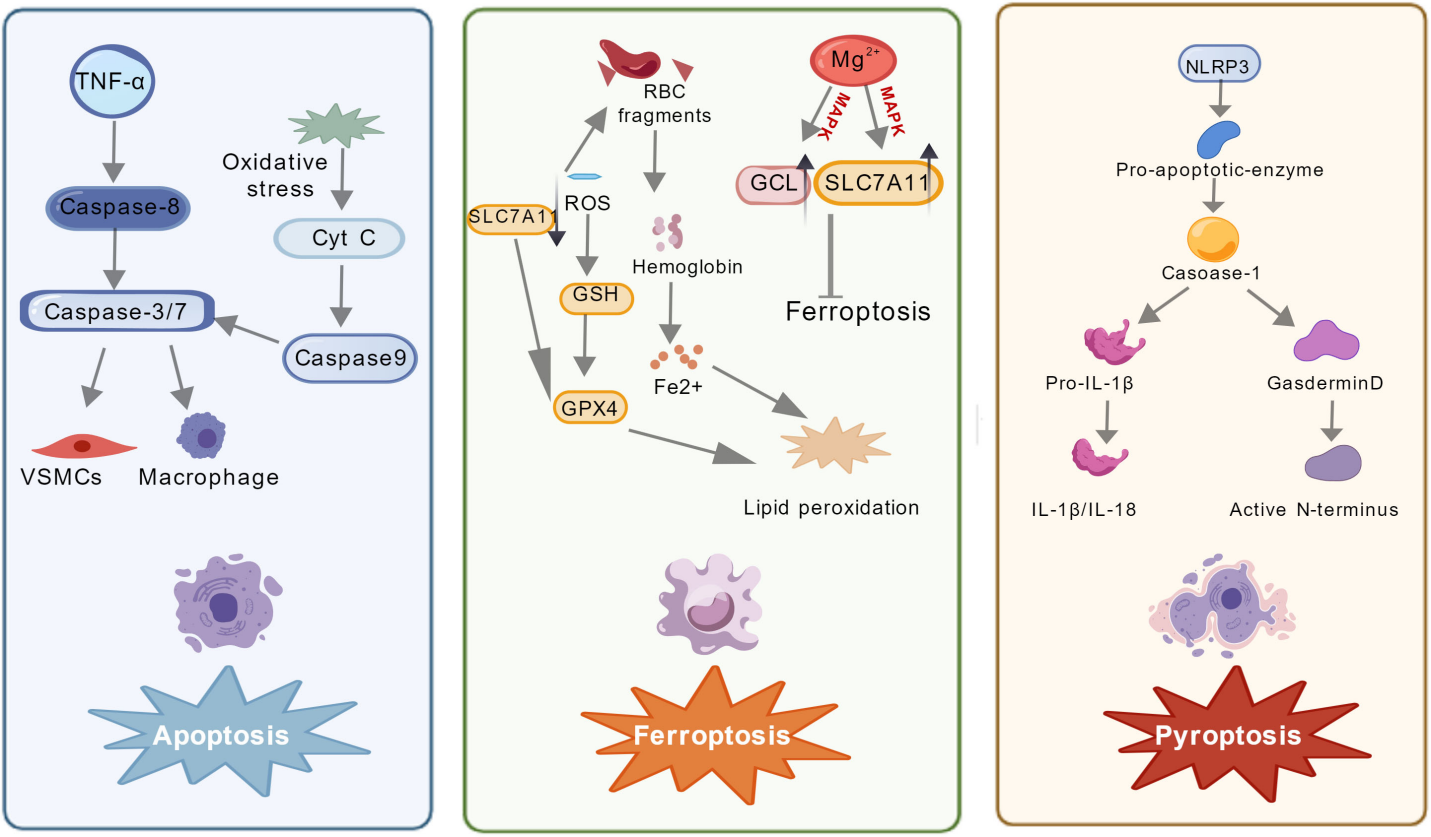
Apoptosis, pyroptosis, and ferroptosis in atherosclerosis. This figure illustrates the molecular mechanisms of three key forms of cell death—apoptosis, pyroptosis, and ferroptosis—in atherosclerosis and their roles in disease progression. In apoptosis, tumor necrosis factor-α (TNF-α) activates Caspase-8, which in turn activates Caspase-3/7. Simultaneously, oxidative stress induces cytochrome c (Cyt C) release, leading to Caspase-9 activation, which also activates Caspase-3/7. These processes ultimately trigger apoptosis in vascular smooth muscle cells (VSMCs) and macrophages. Ferroptosis is initiated by hemoglobin released from red blood cell fragments, generating Fe²^+^, along with elevated reactive oxygen species (ROS). ROS suppresses SLC7A11, reducing glutathione (GSH) synthesis, while also depleting GSH and diminishing glutathione peroxidase 4 (GPX4) activity. Additionally, Mg²^+^ modulates GCL and SLC7A11 via the MAPK pathway, further disrupting GSH metabolism. These events lead to Fe²^+^-mediated lipid peroxidation and ferroptosis. Pyroptosis involves NLRP3 inflammasome activation of Caspase-1, which cleaves Pro-IL-1β to generate the pro-inflammatory cytokines IL-1β/IL-18, while also cleaving Gasdermin D to release its active N-terminal domain, collectively promoting inflammatory cell death.

In the complex pathology of atherosclerosis, the diversity of cell death modes and their subsequent effects are critical determinants of plaque stability and disease progression. Beyond traditional apoptosis, novel forms of programmed cell death—such as necroptosis, pyroptosis, and ferroptosis—have been increasingly uncovered (**Figure 3**). These processes collectively shape the inflammatory microenvironment and plaque fate in atherosclerosis through distinct molecular mechanisms.

#### 2.5.1 Apoptosis

Apoptosis of VSMCs and macrophages is widespread in atherosclerotic plaques and critically influences plaque stability. VSMC apoptosis leads to thinning of the fibrous cap, while macrophage apoptosis impedes lipid clearance; both processes jointly compromise plaque integrity (54). Moreover, impaired clearance of apoptotic cells—specifically, defective efferocytosis—can result in secondary necrosis, releasing abundant pro-inflammatory contents and sharply exacerbating local inflammation (55). Concurrently, apoptotic VSMCs induce the production of stromal cell-derived factor-1α, which recruits circulating mesenchymal stem cells to the lesion site and participates in vascular wall remodeling, thereby linking cell death with repair responses (54). Thus, these interconnected mechanisms collectively illustrate the complex dynamics of apoptosis in atherosclerosis progression.

#### 2.5.2 Pyroptosis

Pyroptosis is a highly pro-inflammatory form of programmed cell death mediated by the gasdermin D protein (56, 57). Its N-terminal domain forms pores in the cell membrane, leading to cell swelling, rupture, and release of potent inflammatory cytokines such as interleukin-1β and IL-18 (58). Activation of the NLRP3 inflammasome is considered a key upstream pathway triggering pyroptosis (59). Recent studies further reveal that in arteriosclerosis obliterans, the metabolite cystine S-sulfate promotes the differentiation of helper T cells 17 and simultaneously induces pyroptosis, synergistically driving disease progression (59). These findings not only emphasize the intricate connections among metabolic abnormalities, immune responses, and pyroptosis in atherosclerosis but also identify potential targets for intervention. Future research may leverage these insights to develop more precise therapeutic strategies.

#### 2.5.3 Ferroptosis

Ferroptosis is an iron-dependent regulatory cell death process driven by lipid peroxide accumulation, characterized by glutathione depletion and loss of glutathione peroxidase 4 activity (60, 61). In atherosclerosis, ferroptosis of endothelial cells disrupts vascular barrier integrity and accelerates lesion progression. Notably, magnesium ions inhibit endothelial ferroptosis and delay atherosclerosis by activating the mitogen-activated protein kinase signaling pathway, upregulating solute carrier family 7 member 11 and glutamate-cysteine ligase expression, while downregulating acyl-CoA synthetase long-chain family member 4 (11). This mechanism not only reveals the cytoprotective role of magnesium but also provides theoretical support for magnesium-based bioabsorbable stents, which may confer additional vascular protection beyond mechanical support (11). Additionally, systemic iron metabolism disorders are closely associated with atherosclerosis risk, further underscoring the importance of maintaining iron homeostasis for vascular health (6). Therefore, targeting the ferroptosis pathway may represent a potential strategy for future cardiovascular disease prevention and treatment.

In summary, apoptosis, pyroptosis, and ferroptosis play pivotal roles in the initiation and development of atherosclerosis through their distinct molecular pathways. These processes interact and form a complex regulatory network of cell death. A deeper understanding of their crosstalk and decisive impact on plaque stability not only expands our knowledge of atherosclerosis mechanisms but also lays a foundation for novel interventions—such as enhancing efferocytosis, inhibiting the NLRP3 inflammasome, or applying magnesium-based materials. Future research should further clarify their specific applications in clinical practice.

### 2.6 Metabolic reprogramming

In atherosclerosis, cells alter their metabolic pathways through metabolic reprogramming to adapt to environmental stress. The metabolic states of vascular and immune cells profoundly influence their functions and phenotypes. Recently, mitochondrial one-carbon metabolism has emerged as a critical pathway, demonstrated to regulate T follicular helper (Tfh) cell differentiation. For example, in transplant atherosclerosis models, CD34^+^ lineage cells differentiate into Tfh cells via this pathway, promoting tertiary lymphoid structure formation and vascular remodeling. Specifically, MTHFD2 enzyme deficiency significantly suppresses Tfh cell differentiation and delays atherosclerosis progression, suggesting that targeting this pathway may offer a new interventional strategy (45). Meanwhile, lactylation modification—an emerging post-translational modification—has been increasingly implicated in atherosclerosis. Integrated single-cell and bulk transcriptome data, combined with machine learning, have identified hub genes such as SOD1, DDX42, and PDLIM1. These genes participate in inflammatory and metabolic processes and may serve as diagnostic markers or therapeutic targets (62). Furthermore, classic metabolic pathways including glycolysis, fatty acid oxidation, and amino acid metabolism contribute to disease progression. For instance, pro-inflammatory macrophage polarization is accompanied by increased glycolytic flux, while endothelial dysfunction correlates with mitochondrial impairment. These metabolic alterations not only regulate energy supply but also drive atherosclerosis through inflammatory cytokine secretion and oxidative stress (2). In conclusion, multiple metabolic pathways intertwine in atherosclerosis and collectively shape the immune and metabolic microenvironment, providing a rationale for developing precision therapies targeting metabolic networks.

### 2.7 Epigenetic regulation

Epigenetic mechanisms regulate gene expression without altering the DNA sequence and serve as a key interface between environmental factors and genetic background. They extensively modulate vascular cell functions and immune responses in atherosclerosis, with histone modifications and non-coding RNAs playing central roles. Specifically, histone deacetylases (HDACs) regulate endothelial function, VSMC phenotypic switching, and inflammatory responses by modifying the acetylation status of histones and non-histone proteins. Aberrant HDAC expression is closely linked to atherosclerosis; for example, HDAC9 overexpression promotes vascular inflammation and plaque formation, whereas HDAC inhibitors ameliorate atherosclerotic lesions in experimental animals (2). Additionally, HDACs influence atherosclerosis progression by regulating cholesterol metabolism and autophagy-related gene expression (63). On the other hand, non-coding RNAs are vital components of epigenetic regulation. Multiple miRNAs—such as miR-133a and miR-30a-3p—and circular RNAs fine-tune various aspects of atherosclerosis by modulating target gene expression. For instance, miR-133a participates in lower extremity atherosclerosis by targeting the RhoA signaling pathway (64), while miR-30a-3p inhibits VSMC proliferation and migration by suppressing ROCK2 expression (50). Concurrently, circular RNAs influence disease progression by acting as miRNA sponges or regulating transcription factor activity (65). Together, these mechanisms unveil the intricate network of epigenetic regulation in atherosclerosis and offer a theoretical basis for developing targeted therapeutic strategies.

## 3 Challenges in the prevention and treatment of atherosclerosis

**Figure 4:**
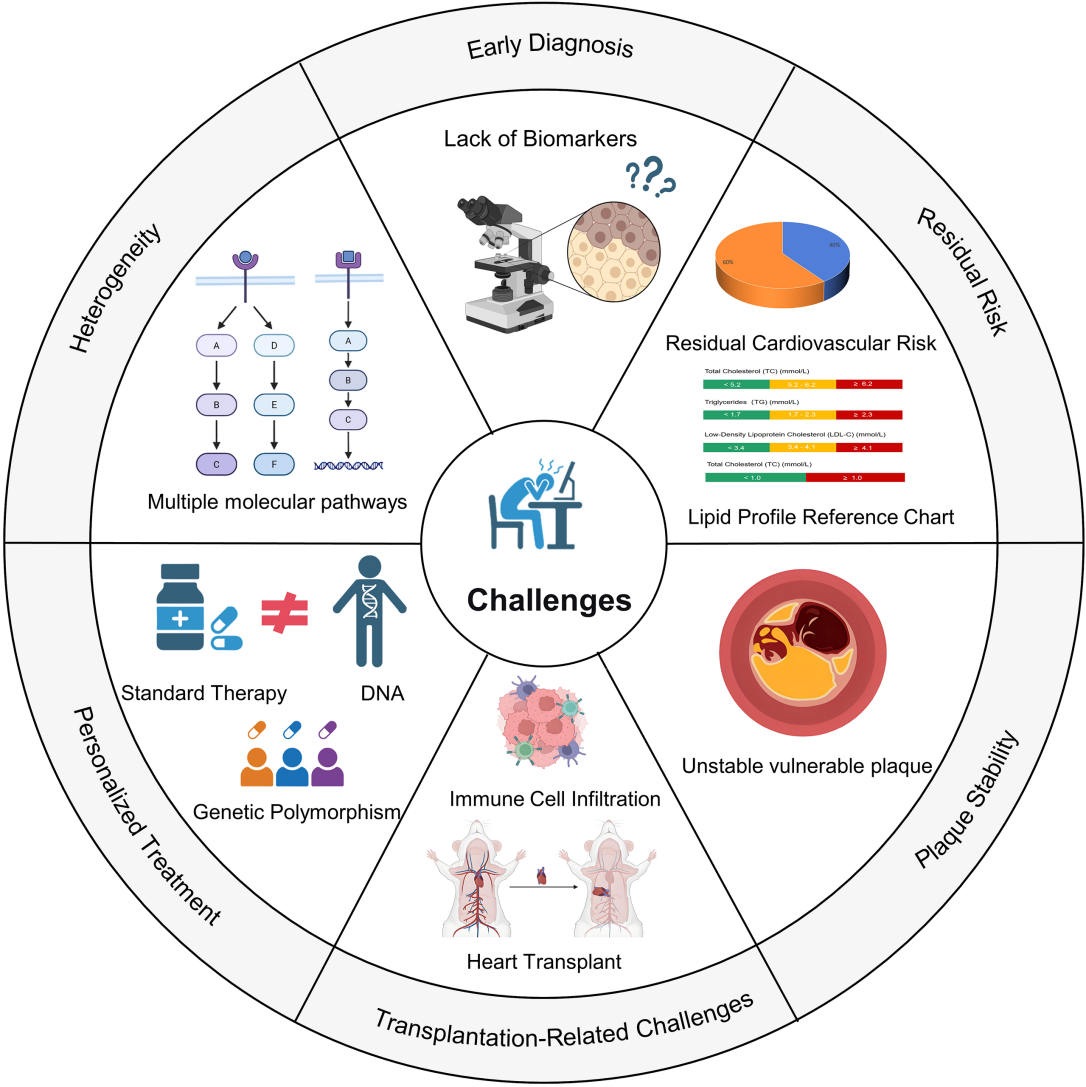
Major challenges in atherosclerosis management. Summary of the principal challenges in preventing and treating atherosclerosis. Key issues include pathogenic heterogeneity among patients and plaques, limitations in early diagnosis and risk assessment, persistent residual cardiovascular risk despite LDL-C control, difficulties in stabilizing vulnerable plaques, lack of personalized treatment strategies, and unique complications in transplant-associated atherosclerosis. Icons and brief descriptions visually represent each barrier to effective clinical translation.

Atherosclerosis is a complex vascular pathology and serves as the primary pathological basis for cardiovascular diseases. Although significant progress has been made in understanding its molecular mechanisms and exploring treatment strategies, clinical prevention and management still face severe challenges (**Figure 4**). These challenges profoundly impact early intervention, therapeutic efficacy, and long-term prognosis, necessitating in-depth analysis and resolution.

### 3.1 Complexity and heterogeneity of pathogenesis

Atherosclerosis is not driven by a single cause but is a complex network disease involving multiple molecular pathways. The dominant pathological mechanisms can vary significantly among patients and even across different plaques within the same individual. For instance, processes such as inflammatory responses, lipid metabolism abnormalities, oxidative stress, calcification, and immune cell infiltration contribute differently across individuals or disease stages (66). This high heterogeneity poses a major obstacle to developing universal therapies. Drugs targeting specific pathways, such as monoclonal antibodies against particular inflammatory factors, may be effective in certain patient subgroups but show limited efficacy in others (16). Therefore, future treatment strategies must shift from a “one-size-fits-all” approach to precision interventions based on molecular subtyping.

### 3.2 Bottlenecks in early diagnosis and risk assessment

Current clinical diagnosis of atherosclerosis still relies heavily on imaging to detect obvious plaque formation or luminal stenosis. By this stage, the disease has often progressed to mid- or late phases, missing the optimal window for intervention. Thus, the lack of highly sensitive and specific early molecular diagnostic markers remains a core bottleneck for early prevention. Although circulating non-coding RNAs (e.g., miRNAs and circRNAs), metabolites, and proteomes are considered promising biomarkers, their translational application faces challenges such as poor reproducibility, lack of standardization, and inconsistent detection techniques (67). Moreover, integrating these emerging markers with traditional clinical risk scores (e.g., Framingham risk score) to construct more accurate risk prediction models is a critical issue requiring resolution (68). Future studies should promote biomarker standardization and model integration using multi-center, large-scale cohorts to enhance early identification and risk assessment of atherosclerosis.

### 3.3 Residual cardiovascular risk

Even after effective control of low-density lipoprotein cholesterol (LDL-C) with statins, patients still face residual cardiovascular risks driven by factors such as inflammation, elevated triglycerides, and dysfunctional high-density lipoprotein. Effectively targeting these non-LDL-C pathways is a central challenge in current lipid-lowering therapy. Studies indicate that persistent inflammatory states (e.g., elevated high-sensitivity C-reactive protein) remain independent predictors of cardiovascular events even when LDL-C levels are controlled (6). Additionally, cholesterol from triglyceride-rich lipoprotein remnants has been independently associated with the development and progression of atherosclerosis (8). Therefore, developing new drugs targeting residual risks—such as inflammation inhibitors (e.g., IL-1β antagonists) and novel triglyceride-lowering agents—and exploring their combination with existing therapies are key future directions.

### 3.4 Challenges in regulating plaque stability

Converting unstable vulnerable plaques into stable ones is critical for preventing acute events such as acute coronary syndromes and strokes. However, current therapies face multiple challenges, including how to precisely suppress intraplaque inflammation while promoting collagen synthesis without exacerbating calcification, and how to balance vascular smooth muscle cell apoptosis and proliferation. Enhancing efferocytosis to clear apoptotic cells, thereby preventing secondary necrosis and amplification of inflammation, is recognized as a central mechanism for plaque stabilization. Furthermore, studies have demonstrated that C-type natriuretic peptide (CNP) promotes an anti-inflammatory macrophage phenotype and augments efferocytosis, leading to a reduction in the necrotic core and increased plaque stability (69). Meanwhile, growing research on novel cell death modes such as ferroptosis and pyroptosis is uncovering new perspectives and potential targets for regulating plaque stability (11). Thus, investigating these emerging mechanisms may provide more diverse and precise therapeutic avenues for cardiovascular disease management.

### 3.5 Lack of personalized treatment

Current guidelines for cardiovascular disease prevention and treatment are primarily based on evidence from large-scale population trials. They lack precision strategies tailored to individual genetic backgrounds, molecular phenotypes, and environmental factors. For example, inter-individual variations in responses to the same lipid-lowering drug may be linked to genetic polymorphisms (e.g., in PCSK9 or APOE genotypes). Advances in single-cell sequencing have revealed remarkable cellular heterogeneity in atherosclerotic lesions, providing unprecedented resolution for understanding individual differences (12). The future challenge lies in translating multi-dimensional omics data—genomics, epigenomics, proteomics, and metabolomics—into clinically applicable decision-making tools to achieve “one-size-fits-one” personalized therapy.

### 3.6 Special challenges in transplant-associated atherosclerosis

Transplant atherosclerosis is a major cause of long-term graft failure after solid organ transplantation (e.g., heart or kidney). Its pathogenesis involves not only traditional risk factors but also complex interactions between persistent alloimmune responses and non-immune factors (e.g., donor organ ischemia-reperfusion injury and immunosuppressant toxicity), making prevention and treatment particularly difficult. Studies show that recipient-derived immune cells and stem cells can infiltrate graft vessels, driving vascular wall remodeling and neointimal hyperplasia (16). Recent research indicates that lymphangiogenesis plays a critical role in mediating host immune cell homing to the graft, and inhibiting the VEGF-C/VEGFR-3 signaling pathway can effectively mitigate transplant atherosclerosis (70). Furthermore, mitochondrial one-carbon metabolism has been shown to regulate T follicular helper cell differentiation, influencing tertiary lymphoid organ formation and thereby contributing to transplant vasculopathy (45). These findings highlight novel mechanisms such as immunometabolism, offering potential targets for managing this challenging complication.

## 4 Potential prevention and treatment strategies and future directions

**Figure 5:**
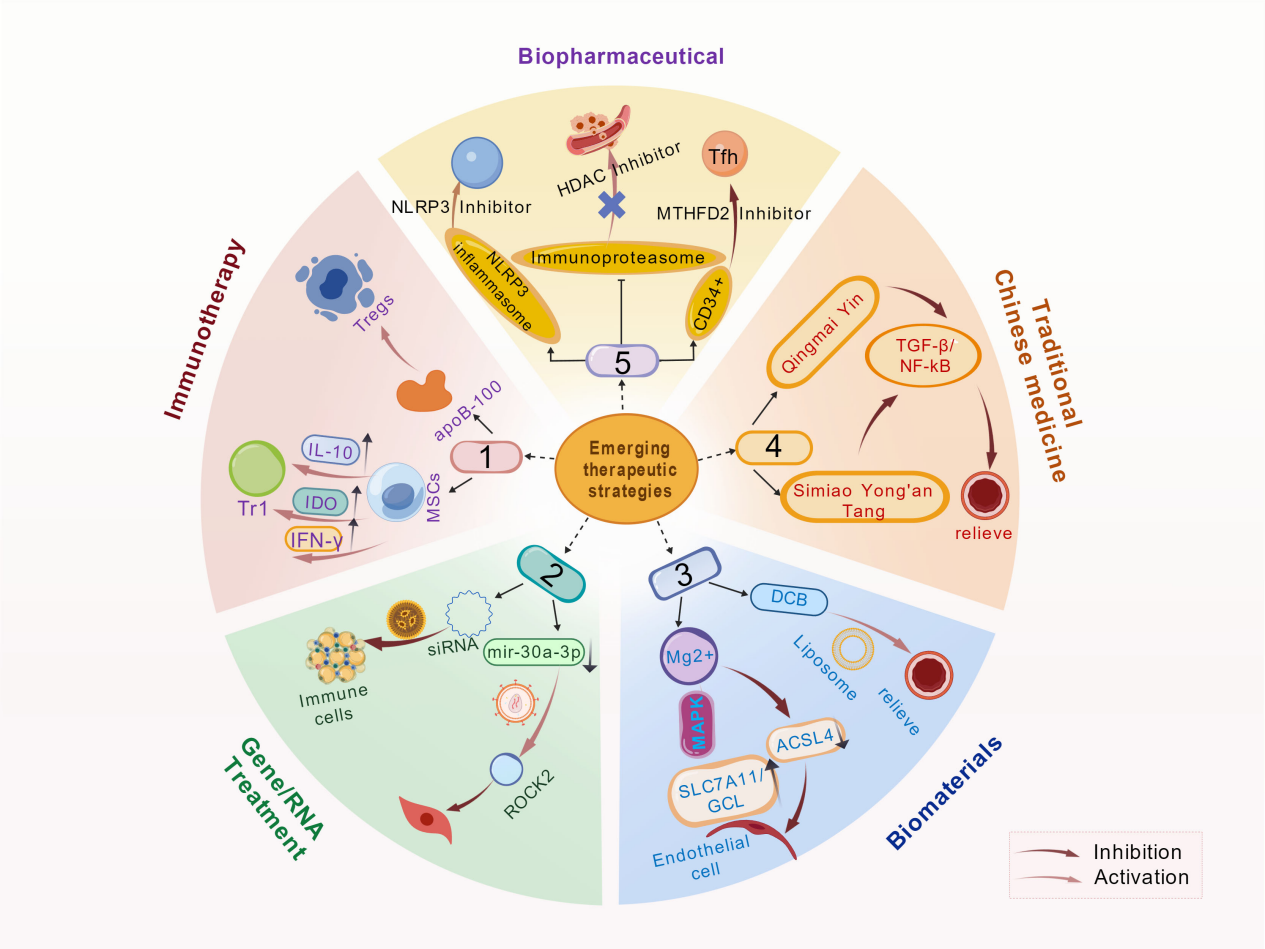
Emerging therapeutic and preventive strategies. This schematic diagram outlines emerging therapeutic strategies for arteriosclerosis, categorized into five areas: Immunotherapy regulates immune cells and cytokines to improve the plaque microenvironment. Examples include Treg/Tr1 cells secreting IL-10 and IFN-γ, and mesenchymal stem cells targeting apoB100. Gene/RNA Therapy uses siRNA or miR-30a-3p to modulate targets like ROCK2, with immune cells aiding nucleic acid delivery. Biomaterials employ carriers such as liposomes and drug-coated balloons, while magnesium ions inhibit ferroptosis via the MAPK pathway. Traditional Chinese Medicine employs formulas like Qingmai Yin to target TGF-β and NF-κB pathways, reducing inflammation. Biopharmaceuticals use inhibitors (e.g., NLRP3, HDAC) to target inflammasomes and epigenetic regulation, and modulate immune cells like Tfh and CD34^+^ cells.

With the deepening understanding of atherosclerosis pathogenesis, prevention and treatment strategies have progressed from traditional risk factor management to a new era of precision interventions targeting specific molecular pathways and immune-inflammatory networks. This progression builds upon established non-pharmacological approaches and conventional pharmacotherapies, now expanding to include novel molecular targets, applications of gene and RNA therapies, innovations in biomaterials and medical devices, modern research on traditional Chinese medicine, and multi-omics-driven precision medicine (**Figure 5**). Together, these developments form a diversified frontier in atherosclerosis management. These advances not only enhance our understanding of the disease but also establish a solid foundation for more efficient and personalized therapeutic approaches, marking a new phase in atherosclerosis prevention and treatment.

### 4.1 Non-pharmacological interventions and lifestyle management

Before describing novel pharmacological strategies, it is essential to acknowledge the foundational role of non-pharmacological management, which possesses robust scientific evidence and demonstrated efficacy. Lifestyle interventions constitute the first-line strategy for primary and secondary prevention of atherosclerosis (1). Regular physical exercise improves vascular endothelial function by enhancing nitric oxide bioavailability and reducing oxidative stress and inflammatory markers such as C-reactive protein (CRP). Dietary modifications, particularly the adoption of Mediterranean-style diets rich in unsaturated fats, fiber, and antioxidants, have been shown to reduce cardiovascular events by improving lipid profiles and attenuating systemic inflammation (1, 68). Smoking cessation represents another critical intervention, as it directly ameliorates endothelial dysfunction and reduces monocyte adhesion and platelet activation. Additionally, weight management through caloric restriction and physical activity not only addresses obesity-related metabolic disturbances but also directly impacts atherosclerotic progression through mechanisms involving adipokine regulation and reduced vascular inflammation. These non-pharmacological approaches provide the essential foundation upon which all pharmacological interventions are built, and their implementation remains crucial throughout the disease continuum.

### 4.2 Conventional pharmacological therapies and their mechanisms of action

Before exploring emerging treatments, a comprehensive understanding of conventional pharmacological therapies is imperative. Statins (HMG-CoA reductase inhibitors) represent the cornerstone of atherosclerotic cardiovascular disease prevention and treatment. Beyond their well-established lipid-lowering effects through inhibition of cholesterol biosynthesis, statins exert multiple pleiotropic effects that contribute to their clinical benefits. These include improvement of endothelial function via upregulation of endothelial nitric oxide synthase (eNOS), anti-inflammatory properties mediated through reduction of CRP and inhibition of leukocyte adhesion molecule expression, stabilization of vulnerable plaques by reducing macrophage content and increasing collagen formation, and potential immunomodulatory effects (2, 8). Despite intensive statin therapy, significant residual cardiovascular risk persists, driven largely by inflammatory pathways and other non-LDL-C factors (6, 8). Other conventional agents include antiplatelet therapies (e.g., aspirin, P2Y12 inhibitors) that prevent thrombotic complications, angiotensin-converting enzyme inhibitors and angiotensin receptor blockers that counter the pro-atherogenic effects of the renin-angiotensin-aldosterone system, and newer lipid-lowering agents such as ezetimibe and PCSK9 inhibitors that further reduce atherogenic lipoproteins through complementary mechanisms (9, 71). These established therapies provide the fundamental pharmacological framework for atherosclerosis management.

### 4.3 Anti-atherosclerotic effects of new drugs

In addition to conventional anti-atherosclerotic pharmacotherapies, emerging hypoglycemic agents—particularly glucagon-like peptide-1 (GLP-1) receptor agonists (GLP-1 RAs) and sodium-glucose cotransporter 2 inhibitors (SGLT2i)—have demonstrated significant cardiovascular protective benefits. These therapeutic effects extend well beyond glycemic control, offering novel opportunities for the integrated management of atherosclerotic cardiovascular disease.

Preclinical studies have elucidated multiple direct anti-atherogenic mechanisms of GLP-1 RAs, including suppression of pro-inflammatory cytokine production (TNF-α, IL-1β, IL-6) in macrophages, inhibition of monocyte adhesion to endothelial cells through downregulation of adhesion molecules, reduction of oxidized LDL-induced foam cell formation, and attenuation of atherosclerotic plaque development in murine models (72–74). These effects appear mediated through GLP-1 receptor-dependent pathways in endothelial cells, with studies demonstrating that GLP-1 RAs improve endothelial function, reduce vascular oxidative stress, and increase NO bioavailability (75, 76). Clinical trials have substantiated these mechanistic insights, demonstrating cardiovascular benefits across diverse patient populations. The SELECT trial showed a 20% reduction in major adverse cardiovascular events (MACE) with semaglutide in overweight or obese individuals without diabetes (77). Similarly, the LEADER (liraglutide), SUSTAIN-6 (semaglutide), and REWIND (dulaglutide) trials demonstrated significant MACE reduction in patients with type 2 diabetes (78, 79). These findings position GLP-1 RAs as a therapeutically important class for atherosclerosis management, particularly in patients with concurrent metabolic diseases.

SGLT2i also demonstrate anti-atherosclerotic effects. Preclinical evidence indicates that SGLT2i exert direct atheroprotective effects by modulating inflammatory pathways, reducing oxidative stress, improving endothelial function, and enhancing plaque stability (80–82). Specifically, empagliflozin and dapagliflozin have been shown to suppress the release of pro-inflammatory cytokines (e.g., TNF-α, IL-6, MCP-1), inhibit NLRP3 inflammasome activation, reduce foam cell formation, and attenuate leukocyte adhesion and migration into the vascular intima (80–82). Additionally, SGLT2i promote autophagy and reduce cellular senescence through pathways involving AMPK, GSK3β, SIRT3, and mTOR, thereby mitigating vascular aging and dysfunction (83, 84). In terms of vascular remodeling, SGLT2i improve endothelial function by increasing nitric oxide bioavailability, inhibiting vascular smooth muscle cell proliferation, and reducing neointimal formation (85, 86). Clinically, SGLT2i have shown benefits across various atherosclerotic manifestations. In coronary artery disease, empagliflozin and dapagliflozin reduce ischemic area, infarct size, and MACE, particularly in patients with prior myocardial infarction or established ASCVD (87, 88). These findings underscore the multifaceted atheroprotective profile of SGLT2i, supporting their use in combination with conventional therapies for comprehensive cardiovascular risk management.

### 4.4 Drug development targeting novel molecular targets

Atherosclerosis management is shifting from conventional risk factor control to precision strategies targeting specific molecular pathways. Recently, drug development focusing on emerging molecular targets has achieved remarkable progress, providing new directions for therapy. As chronic inflammation is a core driver of atherosclerosis, interventions against specific inflammatory pathways have become crucial. For example, NLRP3 inflammasome inhibitors alleviate vascular inflammation by blocking IL-1β and IL-18 maturation (2). Meanwhile, drugs targeting the IL-6 signaling pathway, such as tocilizumab, are under clinical evaluation. On the other hand, AIP1, a key negative regulator of vascular endothelial inflammation, significantly attenuates atherosclerotic lesions by inhibiting the ASK1-JNK/p38 pathway (89). Additionally, targeting the CCL21-CXCR3 axis has been shown to suppress tertiary lymphoid structure formation and delay transplant arteriosclerosis (16). These findings expand our understanding of inflammation in atherosclerosis and provide a basis for novel anti-inflammatory therapies.

In the field of cell death regulation, the inhibition of specific modes of cell death provides novel strategies for improving plaque stability. Ferroptosis inhibitors mitigate atherosclerosis by upregulating the expression of SLC7A11 and GCL and suppressing lipid peroxidation (11). Caspase-1 inhibitors, which target pyroptosis, reduce the release of inflammatory factors and slow plaque progression. Moreover, recent studies demonstrate that modulation of the mitochondrial one-carbon metabolism enzyme MTHFD2 influences T follicular helper cell differentiation and immune responses associated with atherosclerosis (45). These findings underscore the promising therapeutic potential of targeting cell death and metabolic pathways in the treatment of atherosclerosis.

The immunoproteasome, a key component in antigen presentation and immune responses, exhibits synergistic mechanisms when its LMP2 and LMP7 subunits are jointly inhibited. This strategy reduces IgG-secreting and plasma cell numbers while activating the unfolded protein response to suppress antibody production, thereby alleviating transplant arteriosclerosis (48). This dual inhibition outperforms single-subunit targeting, offering a new paradigm for managing immune-mediated vascular pathologies.

Epigenetic regulation also plays a critical role in atherosclerosis progression. Histone deacetylase inhibitors influence disease development by altering chromatin structure and gene expression. Selective HDAC inhibitors like TSA and SAHA suppress phenotypic switching and inflammation in vascular smooth muscle cells (2). Inhibitors targeting specific HDAC subtypes are being developed to balance efficacy and safety. Moreover, scATAC-seq technology enables the analysis of dynamic chromatin accessibility in atherosclerosis, expanding the target space for epigenetic therapies (90). These advances collectively drive atherosclerosis treatment toward more precise and diversified strategies. The discovery of these emerging targets and corresponding drug development signifies the dawn of a precision-targeting era in atherosclerosis therapy. Future efforts should explore synergistic effects among different targeting strategies and validate long-term efficacy and safety through clinical trials.

### 4.5 Immunotherapy and vaccination

Given the immune-inflammatory nature of atherosclerosis, immunomodulatory strategies show broad potential for prevention and treatment. Atherosclerosis is not merely a lipid deposition disease; its pathology involves innate and adaptive immune systems, where aberrant activation of T cells, B cells, and antigen-presenting cells drives chronic vascular inflammation (10). Recently, immunotherapy and vaccination have emerged as novel interventions to halt disease progression by inducing antigen-specific immune tolerance or regulatory cell networks.

In antigen-specific tolerance studies, researchers aim to re-establish immune “ignorance” toward disease-related antigens. For instance, vaccines based on apolipoprotein B-100 (apoB-100) peptides can induce protective antibodies or regulatory T cells (Tregs), specifically suppressing immune-inflammatory responses in atherosclerosis (10). As apoB-100 is a core component of LDL and critical in atherosclerosis initiation, immune tolerance against this antigen effectively blocks subsequent inflammatory cascades and reduces plaque burden in animal models (10). Nanotechnology also offers new platforms for antigen delivery; for example, oral chitosan-DNA nanoparticles encapsulating MHC antigens are taken up by the intestinal lymphatic system, mimicking natural oral tolerance pathways to induce antigen-specific Treg differentiation and suppress intimal hyperplasia in transplant arteriosclerosis models (91). These advances highlight the promise of precise immunomodulation for chronic inflammatory diseases.

Mesenchymal stem cells (MSCs), with their multipotent differentiation capacity and immunomodulatory functions, play key roles in regulating local immune microenvironments through cell therapy. Local infusion of autologous MSCs upregulates IL-10, IFN-γ, and indoleamine 2,3-dioxygenase (IDO), inducing Tr1-like regulatory cells and suppressing transplant arteriosclerosis (92). IDO, a key enzyme in tryptophan metabolism, not only promotes Treg expansion and inhibits effector T cell function but also enhances the immunosuppressive capacity of MSC-induced Tr1-like cells when combined with prostaglandin E2 (PGE2), outperforming MSCs alone in suppressing vascular pathology (93). Further studies indicate that lymphangiogenesis is critical in transplant arteriosclerosis; VEGF-C-mediated lymphatic vessel formation promotes tertiary lymphoid organ development and exacerbates local immune responses, while inhibiting this pathway delays disease progression (70). This provides a theoretical basis for targeting the lympho-immunological axis and opens new directions for optimizing future therapies.

Despite the promise of immunotherapy, challenges remain in antigen specificity, durability of immune tolerance, and individual variability. The safety and standardized delivery systems for cell therapies also require optimization. Future research should focus on precise immunomodulation, integrating multi-omics and personalized medicine to advance clinical translation. In summary, immunoregulatory strategies may open new avenues for atherosclerosis prevention and treatment.

### 4.6 Gene therapy and RNA therapeutics

Gene therapy and RNA therapeutics, as core strategies of precision medicine, offer breakthroughs in atherosclerosis management. These approaches use antisense oligonucleotides, small interfering RNAs (siRNAs), or microRNA (miRNA) mimics/inhibitors to modulate disease-related gene expression, intervening in key pathological processes like vascular smooth muscle cell (VSMC) activation, inflammatory responses, and immune cell migration (94). In gene therapy, viral vector-based delivery systems show significant potential. For example, adenovirus-mediated antisense ERK2 gene therapy specifically inhibits extracellular signal-regulated kinase 2 (ERK2) expression, blocking abnormal VSMC activation and pathological angiogenesis, thereby reducing vascular wall thickening and lumen stenosis in transplant arteriosclerosis models (94). This confirms the central role of the ERK pathway and highlights the advantages of gene therapy in targeting specific molecular mechanisms.

RNA therapeutics represent a powerful strategy for precise gene regulation via non-viral approaches, among which miRNA mimics and antagonists are particularly noteworthy. For example, miR-30a-3p, which is down-regulated in patients with arteriosclerosis obliterans, suppresses vascular smooth muscle cell (VSMC) proliferation, migration, and phenotypic switching by targeting Rho-associated coiled-coil containing protein kinase 2 (ROCK2), thereby delaying neointima formation. In animal studies, lentivirus-mediated delivery of miR-30a-3p significantly attenuated neointimal hyperplasia following carotid artery injury (50). Similarly, miR-142-3p inhibits CD4+ T cell migration and activation via the RAC1/ROCK2 pathway, thereby mitigating perivascular inflammation and offering novel insights into immune-mediated atherosclerosis (95). Beyond miRNAs, siRNA-based therapies have also shown progress in preclinical research. siRNAs directed against proinflammatory cytokines or adhesion molecules effectively suppress endothelial cell activation. Recently, lipid nanoparticle-delivered siRNA targeting VEGF-C successfully inhibited lymphangiogenesis (96). Collectively, these findings underscore the preclinical success of RNA therapeutics and highlight their potential as a promising strategy for treating vascular diseases.

Although gene and RNA therapies hold promise, challenges in delivery efficiency, tissue specificity, and long-term safety remain. Future research should optimize vector design, identify new targets using multi-omics data, and validate efficacy through clinical trials. By integrating gene regulation with immune interventions, this field may open a precision-based, personalized era in atherosclerosis management, indicating the growing role of these therapies in future cardiovascular disease care.

### 4.7 Innovations in biomaterials and medical devices

Advances in materials science and engineering have introduced new strategies for atherosclerosis prevention and treatment. Magnesium-based absorbable stents exemplify “drug-device combination” therapy, overcoming limitations of permanent metal stents that may cause chronic inflammation or restenosis. These stents provide initial mechanical support and gradually degrade *in vivo*, releasing magnesium ions with direct anti-atherosclerotic effects. Recent studies show that magnesium ions activate the MAPK pathway, inhibit ERK protein dephosphorylation, and upregulate solute carrier family 7 member 11 and glutamate-cysteine ligase expression, significantly suppressing ferroptosis in vascular endothelial cells (11). This mechanism, validated in multiple animal models (mice, rats, rabbits), demonstrates that magnesium-based stents not only restore blood flow but also actively delay atherosclerosis by regulating cell death and lipid metabolism.

In early screening and dynamic risk management, wearable devices address the limitations of conventional vascular imaging in continuous monitoring. For example, flexible ultrasonic sensor arrays with automatic phase calibration conform comfortably to neck skin, enabling high-resolution imaging and long-term continuous monitoring of carotid arteries. This allows non-invasive, real-time assessment of vascular wall thickness, plaque formation, and elasticity changes (97). Drug-coated balloons and targeted delivery systems also show potential; paclitaxel-coated balloons combined with separable microneedles enable sustained drug release at lesion sites, improving efficacy and reducing restenosis risk (98). Future integration of wearables with AI algorithms and bioactive materials will make atherosclerosis management more precise, dynamic, and personalized, offering comprehensive patient care.

### 4.8 Lifestyle interventions and multimodal management

Beyond lifestyle and pharmacological interventions, innovations in biomaterials and medical devices provide new strategies for atherosclerosis control. Recent studies on atherosclerosis and transplant arteriosclerosis reveal the potential of advanced material-based therapies. In transplant arteriosclerosis, lymphatic vessel formation begins at anastomotic sites and is driven by fibroblast-derived VEGF-C; inhibiting this pathway significantly reduces lymphangiogenesis, neointima formation, and adventitial fibrosis (70). This suggests that developing devices with localized VEGF-C inhibitor coatings could offer new approaches for preventing transplant arteriosclerosis. Meanwhile, biodegradable magnesium alloy stents show promise; magnesium ions activate the MAPK pathway, upregulate SLC7A11 and GCL expression, inhibit endothelial cell ferroptosis, and downregulate ACSL4 to modulate lipid metabolism, delaying atherosclerosis in various animal models (11). This lays the foundation for next-generation stents with both mechanical support and active anti-atherosclerotic functions.

Drug-coated balloons and stents continue to evolve. For lower extremity arteriosclerosis obliterans, drug-coated devices locally release anti-proliferative agents, significantly improving revascularization outcomes in femoropopliteal artery lesions (99). Additionally, nanoscale targeting systems like hyaluronic acid-modified rapamycin liposomes specifically target the lymphatic system, more effectively modulating immune responses and reducing atherosclerotic lesions (100). These innovations expand treatment options and enable precise intervention in molecular mechanisms. With advances in materials science, drug delivery, and disease mechanism insights, intelligent devices integrating mechanical support, localized drug delivery, and biological regulation will lead future directions in atherosclerosis management.

### 4.9 Modern research on traditional Chinese medicine

Traditional Chinese medicine (TCM) compounds, such as Qingmai Yin and Simiao Yong’an Tang, demonstrate significant efficacy in improving arteriosclerosis obliterans in clinical practice, highlighting TCM’s holistic “multi-component, multi-target” regulatory advantages. Modern pharmacological studies gradually reveal their mechanisms, showing that these compounds modulate key signaling pathways like TGF-β/Smad and NF-κB, improving lipid metabolism disorders and suppressing vascular inflammation to delay atherosclerosis (101, 102). For example, active components in Qingmai Yin inhibit the NF-κB pathway to reduce inflammatory factor release while regulating lipid metabolism-related gene expression, synergistically exerting anti-atherosclerotic effects (101). TCM monomers like Kangle Xin specifically inhibit the PDGFR-β pathway, blocking VSMC phenotypic switching and thereby suppressing abnormal proliferation and migration to delay vascular remodeling (7). This provides new ideas for targeted therapy.

TCM also demonstrates unique value in modulating immune-inflammatory microenvironments. Certain TCM components can influence T cell differentiation and lymphangiogenesis, thereby regulating immune responses in transplant arteriosclerosis. This aligns with the TCM theory of “removing stasis and unblocking collaterals”. Future studies should integrate systems biology and network pharmacology to elucidate synergistic mechanisms of TCM compounds. Clinical efficacy should be further validated through evidence-based medicine. Such efforts will offer multidimensional strategies for atherosclerosis management and advance personalized therapy and drug development (7, 101). Together, these advances highlight the growing potential of TCM in modern medicine.

### 4.10 Precision medicine and novel biomarkers

Precision medicine shows great promise in atherosclerosis management, focusing on integrating multi-omics data—genomics, proteomics, metabolomics, and single-cell sequencing—to build individualized risk prediction models and open new avenues for early warning and precise intervention. Single-cell sequencing technologies have propelled research forward; for example, in transplant arteriosclerosis models, they reveal dynamic cellular population evolution and identify the CCL21/CXCR3 signaling axis as critical for immune cell chemotaxis (16). Simultaneously, mitochondrial one-carbon metabolism influences CD34^+^ lineage cell differentiation into T follicular helper cells and tertiary lymphoid organ formation, providing new perspectives on atherosclerosis immunology (45). These findings deepen our understanding of pathogenesis and lay the groundwork for targeted therapies.

In biomarker discovery, machine learning algorithms enhance the efficiency of identifying diagnostic and prognostic markers for atherosclerosis. Lactylation-related genes exhibit specific expression profiles in carotid atherosclerotic tissues, with SOD1, DDX42, and PDLIM1 identified as core genes, offering potential therapeutic targets (62). Metabolomic analyses further reveal that cysteine-S-sulfate exacerbates atherosclerosis by promoting TH17 cell differentiation and pyroptosis (59). Based on these markers, clinical strategies are moving toward personalization; for instance, polymorphisms like ALKBH5 rs671 and MTHFR rs1801133 can be targeted as independent risk factors for multiple atherosclerosis (103). Additionally, remnant cholesterol is independently associated with disease progression, indicating that high remnant cholesterol levels increase atherosclerosis risk even when LDL cholesterol is controlled (8). These discoveries enhance our molecular understanding and support the development of precision medicine strategies.

Despite its promise, precision medicine faces challenges in integrating multi-omics data, validating novel biomarkers clinically, improving machine learning model generalizability, and translating individualized strategies into practice. Future research must address these key issues to achieve truly precise and personalized atherosclerosis prevention and treatment.

## 5 Summary and outlook

Atherosclerosis is a complex chronic vascular disease. Its development involves interactions across multiple levels, including disordered lipid metabolism, immune-inflammatory responses, cellular dysfunction, and epigenetic regulation. Recent studies have established the central role of low-density lipoprotein cholesterol (LDL-C) accumulation in the vascular wall. Moreover, remnant cholesterol has been identified as an independent risk factor significantly associated with atherosclerosis progression (8). In terms of immune inflammation, lymphangiogenesis drives immune cell infiltration and vascular remodeling via the VEGF-C signaling pathway, thereby promoting transplant arteriosclerosis. Inhibiting this pathway can delay the process (70). Additionally, dysregulated iron metabolism contributes to atherosclerosis by promoting ROS generation and cellular injury (6). Epigenetic regulatory mechanisms, such as histone deacetylases (HDACs), also modulate vascular cell gene expression, influencing inflammatory responses and fibrosis (2). Together, these mechanisms form a complex molecular network, offering multidimensional insights into the disease.

**Figure 6:**
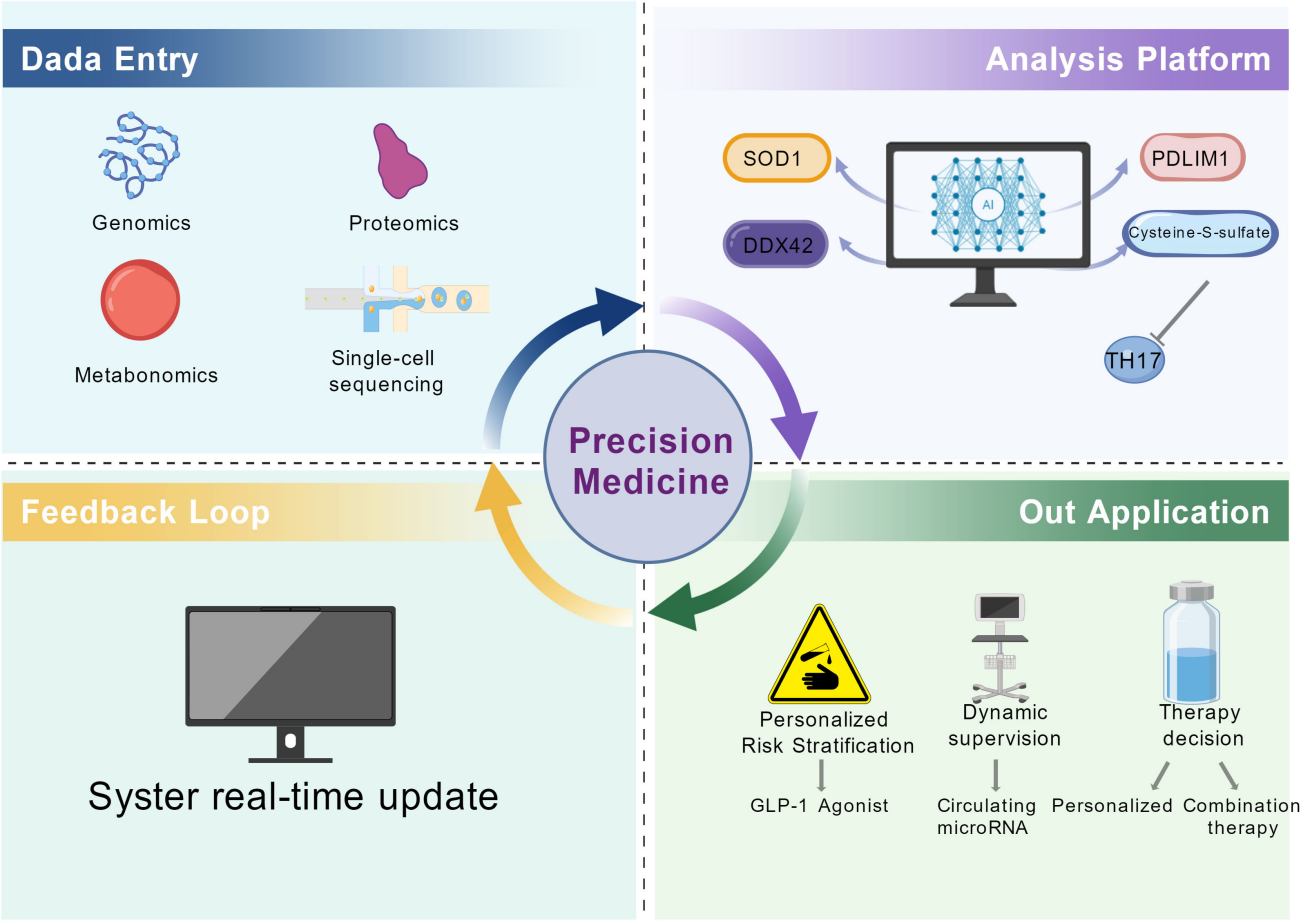
Precision medicine framework for atherosclerosis. This schematic diagram, centered on “Precision Medicine,” outlines a four-module implementation pathway for precision medicine in atherosclerosis. The process begins with the Data Entry module, which integrates multi-omics (genomics, proteomics, metabolomics) and single-cell sequencing data to establish a high-resolution biological information foundation. Next, the Analysis Platform employs artificial intelligence to deeply mine the data, identifying key molecular markers such as SOD1, DDX42, PDLIM1, and cysteine-S-sulfate, and elucidating their regulatory relationships with Th17 cell function. In the Output Application phase, the analytical results are translated into personalized clinical practices, including personalized risk stratification to guide precise use of medications such as GLP-1 receptor agonists, dynamic monitoring of disease progression via biomarkers like circulating microRNAs, and the development of personalized combination therapies. Finally, the Feedback Loop module enables continuous optimization of precision medicine models and strategies through system real-time updates, forming a closed-loop iterative framework that incorporates clinical outcome data back into the analysis platform.

A deeper, mechanistic dissection of the molecular networks is urgently needed. Future research will first focus on deepening the understanding of molecular mechanisms using systems biology. Single-cell sequencing, spatial transcriptomics, and multi-omics integration will help construct dynamic molecular maps across individuals and disease stages (**Figure 6**). For instance, single-cell RNA sequencing has revealed cellular heterogeneity and interaction networks in transplant arteriosclerosis (16). Establishing unified human multi-omics databases for atherosclerosis, combined with artificial intelligence, will help identify key driver pathways and cross-organ interactions. Such systemic insights will strongly support target discovery and intervention strategies, advancing precision diagnostics and treatment. Although single-cell sequencing and multi-omics technologies have begun to reveal cellular heterogeneity and potential driver pathways, substantial gaps exist in functionally validating these discoveries and understanding their dynamic interplay. Future work must move beyond correlation and establish causality; for instance, by employing CRISPR-based screening or organoid models to validate the functional roles of newly identified cell states and signaling pathways, such as those in VEGF-C-driven lymphangiogenesis or HDAC-mediated epigenetic regulation (104). A key challenge is to integrate multi-omics data across temporal and spatial dimensions to build predictive models of plaque progression and stability, rather than static snapshots.

In early diagnosis and dynamic monitoring, technological innovations are moving atherosclerosis management toward earlier stages. This includes developing highly sensitive biomarkers and advanced non-invasive imaging for ultra-early warning and treatment evaluation. For example, arterial stiffness assessment based on pulse wave velocity and ankle-brachial index is widely used clinically (8). Emerging liquid biopsies, such as circulating microRNAs, plaque-targeted molecular imaging, and wearable ion sensors (105), are improving diagnostic accuracy and convenience. The maturation and integration of these technologies will enable real-time dynamic monitoring, providing timely and comprehensive information for clinical decisions, thereby enhancing early screening coverage and diagnostic accuracy. The translation of novel biomarkers and imaging techniques into clinical practice faces significant hurdles. The specificity and clinical utility of many proposed biomarkers, such as circulating microRNAs, require rigorous validation in large, prospective, and diverse cohorts to establish standardized cut-off values. For molecular imaging, the development of next-generation probes with higher specificity for vulnerable plaques and the ability to quantify therapeutic response is a critical next step. Furthermore, the integration of data from wearables, liquid biopsies, and imaging into unified risk prediction algorithms represents a major, yet essential, computational and clinical challenge.

Regarding treatment strategies, atherosclerosis management is expanding from conventional single-drug therapies to innovative approaches and combination regimens. These include small-molecule drugs like MTHFD2 inhibitors, biologics such as anti-IL-17 antibodies, cell and gene therapies, and novel vaccines. For example, immunomodulatory therapies have shown potential in transplant arteriosclerosis models (13). Future efforts should promote multi-modal combination strategies to overcome current therapeutic limitations. Driven by deeper molecular insights, prevention and treatment strategies for atherosclerosis are shifting from traditional lipid-lowering approaches to multi-target, individualized, and immunomodulatory integrated models. For example, immune-inflammatory targeting strategies, such as anti-cytokine therapies and apolipoprotein B-100 vaccines, have shown promise in preclinical models (10). Meanwhile, precision medicine integrates genetic, metabolic, and imaging data to enable individualized risk stratification and intervention, such as lipoprotein(a) [Lp(a)]-based risk management and treatment decisions (71). These advances collectively promote more systematic and targeted approaches for future clinical practice. The promise of multi-target and immunomodulatory approaches is tempered by several unresolved issues. A primary area for investigation is determining the optimal combinations and sequences of therapies (e.g., lipid-lowering plus anti-inflammatory) to achieve synergistic efficacy without compromising safety. The long-term safety and off-target effects of emerging modalities, including gene therapies and vaccines, must be thoroughly evaluated in relevant disease models. Additionally, the field must address the challenge of “target redundancy” in complex biological networks by identifying key nodal points that are less susceptible to compensatory bypass mechanisms.

Furthermore, the concept of precision medicine will fully permeate atherosclerosis risk prediction and treatment implementation. Based on genetic background, molecular subtypes, and real-time physiological parameters, dynamic risk models and highly individualized interventions will be developed. For instance, Pelacarsen for high Lp(a) individuals and GLP-1 receptor agonists for diabetic patients with atherosclerosis (106) demonstrate the promise of targeted therapy. As more biomarkers and subtyping tools emerge, personalized medication and dynamic regulation will become clinical standards. This progression will ultimately shift atherosclerosis management from a “one-size-fits-all” model to a “tailor-made” approach. The implementation of precision medicine necessitates a more granular understanding of disease endotypes. Current efforts, such as targeting Lp(a) with Pelacarsen, are a promising start, but future research needs to define atherosclerosis subtypes based on integrated molecular, imaging, and clinical data. This requires building large-scale, multi-ethnic biobanks with deeply phenotyped data. Crucially, clinical trials must evolve to test the efficacy of stratified therapies in these pre-defined subpopulations, moving away from the one-size-fits-all paradigm.

Although current prevention and treatment face challenges such as target redundancy, individual heterogeneity, and long-term safety, ongoing advances in understanding molecular mechanisms and rapid biotechnological developments—including gene editing and nanomedicine delivery—hold promise for early blockade or even reversal of atherosclerosis in the near future. By strengthening multidisciplinary collaboration and promoting the clinical translation of innovative strategies, the global burden of this major chronic disease may be fundamentally alleviated. To overcome these challenges, strengthening interdisciplinary collaboration among molecular biologists, clinical researchers, data scientists, and bioengineers will be paramount. The concerted application of advanced tools—from gene editing and nanomedicine for targeted delivery to artificial intelligence for data integration—holds the potential to transform atherosclerosis management from a reactive to a proactive, preemptive, and personalized endeavor, ultimately alleviating its global burden.
